# Dietary Probiotic Compound Improves Reproductive Performance of Porcine Epidemic Diarrhea Virus-Infected Sows Reared in a Japanese Commercial Swine Farm under Vaccine Control Condition

**DOI:** 10.3389/fimmu.2017.01877

**Published:** 2017-12-22

**Authors:** Takio Inatomi, Masaaki Amatatsu, Gustavo A. Romero-Pérez, Ryo Inoue, Takamitsu Tsukahara

**Affiliations:** ^1^Inatomi Animal Hospital, Tokyo, Japan; ^2^TOA Pharmaceutical, Tokyo, Japan; ^3^Kyoto Institute of Nutrition & Pathology, Kyoto, Japan; ^4^Laboratory of Animal Science, Kyoto Prefectural University, Kyoto, Japan

**Keywords:** probiotic compound, porcine epidemic diarrhea virus, reproductive performance, sow, milk production, vaccine administration

## Abstract

Lactogenic immunity transferred to piglets after inoculation of a live vaccine to pregnant sows was proved limited to control porcine epidemic diarrhea (PED). Hence, here we evaluated the efficacy of administration of a probiotic compound containing *Bacillus mesentericus, Clostridium butyricum*, and *Enterococcus faecalis* together with a commercial live-attenuated PED vaccine (Nisseiken PED Live Vaccine, Nisseiken, Tokyo, Japan) to improve the health and reproductive performance of PED-infected sows. Twenty pregnant sows in a PED-positive farm were equally divided into probiotics-administered (VP) and control (VC) sow groups. A commercial live-attenuated vaccine was injected as per the manufacturer’s instruction. The probiotic compound (15 g/day) was orally administered to VP from 6 weeks pre-parturition to 7 days post-parturition (ppd7). VP had a significantly higher body weight at ppd7 than VC (191 vs 186 kg; *P* < 0.05). At day 3 post-parturition (ppd3) (4.18 vs 3.63 kg/day) and ppd7 (5.14 vs 4.34 kg/day), milk produced by VP was significantly (*P* < 0.05) greater than that by VC. Total immunoglobulin (Ig)A and IgG concentrations at day 0 were significantly (*P* < 0.05) higher in whey of VP (1.9 and 6.6 g/dL, respectively) than in that of VC (1.7 and 6.1 g/dL, respectively). However, total IgG concentration in whey of VP and VC at ppd3 and ppd7 did not differ. Antibody titer was significantly higher at day 0 in serum of VP than it was that of VC (60 vs 37 in geometric mean; *P* < 0.05). Likewise, the antibody titer in whey of VP and VC was found to be similar at day 0 (416 vs 208 in geometric mean; *P* = 0.13). Consequently, VP had fewer days between weaning and return to estrus than did VC (7 vs 10 days; *P* < 0.05). Moreover, piglets of VP had a significantly (*P* < 0.05) higher litter weight at birth (9,252 g/litter) and a lower mortality (12%) during suckling than those of VC (8,686 g/litter and 28%, respectively). In summary, probiotic-supplemented, PED-vaccinated sows were healthier, transferred PED-specific antibodies *via* colostrum to piglets, had greater litter weight at birth, and reduced mortality during suckling.

## Introduction

Porcine epidemic diarrhea (PED) is an enteric disease that causes severe economic losses to the pig industry worldwide (1). PED was first recognized in the UK and quickly spread to other European countries (2, 3). In the following years, PED was detected in many Asian countries, including Japan (4), where it re-emerged in 2013 and caused approximately 1,000 outbreaks in 39 of 47 prefectures (3). Intriguingly, the PED strains emerged in Asia are quite distinct from those previously reported (5), as they cause more deleterious effects on all pigs regardless of age (6).

Porcine epidemic diarrhea virus is an enveloped, single-stranded RNA virus belonging to the group 1 of the genus *Coronavirus* (7). PED is characterized by watery diarrhea, dehydration, vomiting, anorexia, and reduced appetite in pigs of all ages. For newborn piglets, mortality caused by PED is close to 100% (8, 9), whereas for suckling piglets mortality can be as high as 80% (10). In addition, PED can seriously diminish the production performance of surviving animals (11). Interestingly, the deleterious effects of PED on reproductive performance of gilts and sows depend on the pregnancy stage during which they contract the disease (12). For example, sows infected with PED during the first 30 days of pregnancy have decreased farrowing rates, increased abortion rates, and more mummified fetus per litter, whereas females infected with PED during 91–120 days of pregnancy have more stillbirth piglets per litter (12). Although depressed milk secretion has been previously reported in PED-infected sows (9), the extent of the effect of this viral infection on lactating sows, including passive immunity or the health status and survival rate of suckling piglets, is yet to be fully investigated.

In recent years, vaccines have been developed in an attempt to eradicate PED (13–16). In theory, pre-parturition vaccination of sows putatively permits enteric sensitization to antigens, after which immunoglobulin (Ig)A immune cells are transferred to the mammary gland and secrete antibodies (17). As a result, passive immunity against PED is putatively conferred by the sow to suckling piglets *via* colostrum and milk (17, 18). In fact, however, lactogenic immunity by live vaccines has been proven only somewhat efficacious in providing protection to piglets against PED (19). For example, PED infections have recently re-emerged in Japan (3), even though, following infection outbreaks, live vaccines were administered to pigs in this country. This lack of effectivity is likely due to either strains from live vaccines producing more infectious mutations in the wild (20) or vaccine strain sequences having limited compatibility with those of wild types (21). It is, therefore, imperative to find antiviral agents that act as adjuvant to existing vaccines and help increase their effectivity. Recently, a probiotic bacteria fused with PED virus core neutralizing epitope antigen was developed to use an anti-PED vaccine (22). In mice, oral administration was the effective strategy for this vaccine, targeting mainly mucosal dendritic cells in the intestine and stimulating PED virus specific immunity. In contrast, efficacy of this oral vaccine for pigs is still obscure, because no validation study for this species has been conducted yet.

Administration of probiotics such as lactic acid bacteria (LAB) has been recognized as a viable alternative to antiviral medication for treating viral infections. For example, viable *Lactobacillus acidophilus* and *Lactobacillus reuteri* have been shown to protect experimental models against viral strains such as human rotavirus (HRV) by improving total intestinal IgA-releasing cell immune responses, as well as total serum IgM, and intestinal IgM and IgG titers (23). Similarly, colonization of the gut of neonatal gnotobiotic pigs with probiotic strain such as *Lactobacillus rhamnosus* strain GG and *Bifidobacterium animalis* ssp. *lactis* BB-12 resulted in significantly lower fecal scores and reduced HRV shedding concentrations but increased intestinal IgA HRV antibody concentrations and HRV-specific IgA antibody-releasing cell numbers in infected animals (24). Likewise, in our premises we demonstrated that a cell preparation of *Enterococcus faecalis* strain EC-12, which is a heat-killed LAB, protected weaning piglets from rotavirus infection (25) and stimulated luminal IgA secretion in young calves (26) and chicks (27). Recently, Sirichokchatchawan et al. (28) demonstrated that live LAB could reduce PED infectivity *in vitro*. These data highlight the potential that probiotics and immunogenics may have to enhance lactogenic immunity and the efficacy of live vaccines administered to farm animals. In a separate study, we demonstrated that a probiotic compound containing *Bacillus mesentericus, Clostridium butyricum*, and *E. faecalis* prevented colibacillosis in weaning piglets (29). We also reported that twofold concentration of total IgA was detected in the ileum in piglets when this probiotic compound was administered. Finally, we also reported in a different study that this three-probiotic strain compound induced twofold concentration of total IgA in the ileum of chicks affected by coccidian infection (30).

The aim of the present study was to evaluate the efficacy of the aforementioned probiotic compound mixed with peptide–zinc to improve the health and reproductive performance of PED-infected lactating sows when administered along with a PED vaccine injection in Japan.

## Materials and Methods

### Probiotics

Probiotic product BIO-THREE PZ (TOA Pharmaceutical Co. Ltd., Tokyo, Japan) containing a mixture of bacterial strains *B. mesentericus* TO-A (1 × 10^6^ CFU/g), *C. butyricum* TO-A (1 × 10^6^ CFU/g), and *E. faecalis* T-110 (1 × 10^8^ CFU/g) in a peptide–zinc compound (10 mg/g) was used in this study.

### Farm

The present work was carried out in a commercial swine farm in Kyushu region of Japan. The farm operates a farrow-to-finish business and has approximately a stock of 900 sows (Landrace x Large white). For the present work, all sows were impregnated by Duroc boars. Experiments were carried out between April and May 2014. Sows ate commercial feed during gestation (Shuton-B; Minami Nihon Kumiai Siryo, Kagoshima, Japan) or lactation (Shuton-Lactation; Minami Nihon Kumiai Siryo) period. The diet was free from intestinal microbiota modifiers, such as antimicrobials and probiotics.

A preliminary enteropathogen survey of the farm showed that none of the following diseases were detected in sows and suckling piglets: rotavirus, transmissible gastroenteritis virus, *Clostridium perfringens*, enterotoxigenic *Escherichia coli, Salmonella* sp., *Brachyspira hyodysenteriae, Lawsonia intracellularis*, or classical swine fever virus. Detection of other pathogens such as porcine reproductive and respiratory virus and porcine circovirus type 2 was positive, but not active during this study. The infection rate of these two viruses in the farm was constant at about 40%, according to the survey of 10 randomly selected sows conducted every 4 months.

Infection outbreaks of PED virus occurred in December 2013 in the region where the farm was located. As a consequence, an infection screening was conducted at the farm by an independent livestock hygiene center. The screening results showed that all sows used in the present study were indeed naturally infected with PED virus.

### Experimental Design

The present experiment was approved by the ethical committee at Inatomi Animal Hospital (approval number 09121018). The experiment was carried out between May 2014 and June 2014. Twenty pregnant sows were equally divided and allocated to either a probiotic-administered group (VP) or a control group (VC) with a similar mean parity (= 3.1). Six weeks before parturition to 1 week after parturition, 15 g/day of the probiotic compound was orally administered to sows in the VP group, whereas 15 g/day of a standard, probiotic-free diet was given to sows in the VC group. A commercial live-attenuated vaccine (Nisseiken PED Live Vaccine, Nisseiken, Tokyo, Japan) containing PED virus strain P-5V (seed)^1^ was injected to all sows (10^4.5^ TCID_50_/head) 6 and 2 weeks before parturition. Each sow fostered nine neonates throughout the experiment. Some piglets died during the experiment. Clinical signs included watery yellowish diarrhea and dehydration. An autopsy showed that their small intestine was thin and flaccid. A qualified clinical veterinarian (Dr. Takio Inatomi) diagnosed that piglets died from PED infection.

### Sample Collection and Analysis

Blood was collected from the jugular vein of sows 14 days prior to parturition and 0 and 7 days post-parturition. Milk secretion was determined by a general method described in the Standard Methods of Evaluation of Reproductive Performance compiled by the Japan’s Pork Producers Association.^2^ Daily volume of milk secretion of all sows was measured at days 3 and 7 post-parturition. A portion of milk was collected at days 0, 3, and 7 post-parturition. In addition, the total body weight of neonates was repeatedly measured immediately prior to and after daily suckling. When piglets of the experimental sows died during lactation, healthy and similar-age foster piglets were introduced from other sows so that all piglets ingested a similar amount of maternal milk.

### Protein and Fat Percentage in Milk

Protein and fat percentages in milk at days 0 and 7 post-parturition were determined by a milk analyzer (MilkoScan™ FT1; FOSS, Eden Prairie, MN, USA).

### Immunologic Parameters in Whey

Whey was collected from milk after centrifugation (13,000 × *g*, 30 min, 4°C). Serum was collected from blood after centrifugation (1,200 × *g*, 20 min; room temperature). Total IgA or G concentrations were measured by a commercial ELISA kit (Porcine IgA or IgG ELISA Quantitation Set; Bethyl, Montogomery, TX, USA). The determination method was as previously described elsewhere (31). A portion of whey was sent to the Nansatu Livestock Hygiene Center (Kagoshima, Japan), and neutralized antibody titer against PED in whey and serum was determined by a general method as previously described elsewhere (10, 32).

### Statistical Analyses

Either the Student’s or the Welch’s *t*-test was applied to analyze differences between mean values in all parameters. Values are shown as mean ± SE. Differences between mean values were considered significant at *P* < 0.05 and a tendency to be significant at *P* < 0.1 in all statistical analyses. All calculations were made using Statcel3 (OMS, Tokyo, Japan) as add-in application for Microsoft Excel^®^ (Microsoft, Seattle, WA, USA).

## Results

### Feed Intake

**Figure 1:**
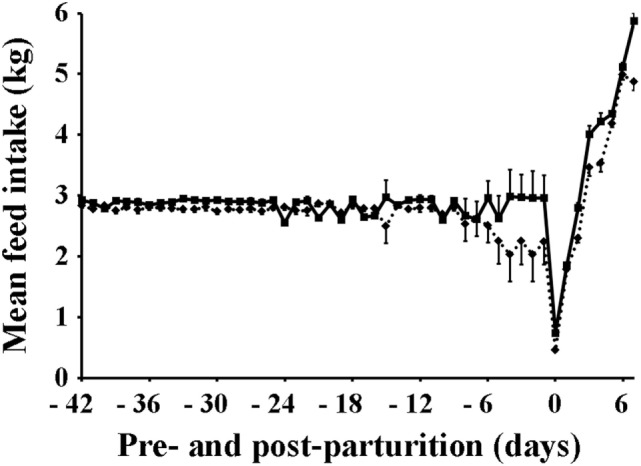
Mean daily feed intake of sows. Squares, solid line: sows supplemented with probiotic compound and administered with a commercial live porcine epidemic diarrhea vaccine. Rhomboids, dotted line: control sows (vaccine only, no probiotics).

The mean feed intake of sows is shown in Table 1 and Figure 1. Sows vaccinated against PED virus and supplemented with a dietary probiotic compound had a significantly higher (*P* < 0.001) feed intake before (2.85 kg) and after parturition (3.62 kg) compared with sows vaccinated against PED virus but receiving no dietary probiotic supplementation (2.69 and 3.20 kg, respectively).

**Table 1:** Mean feed intake of sows during the pre- and post-parturition periods in the experiments (kg/day).

Period (days)	VC	VP	*t*-Test *P* value
Pre-parturition (−42 to −1)	2.69 ± 0.02	2.85 ± 0.03	<0.001
Post-parturition (0–7)	3.20 ± 0.04	3.62 ± 0.04	<0.001

### Body Condition

**Figure 2:**
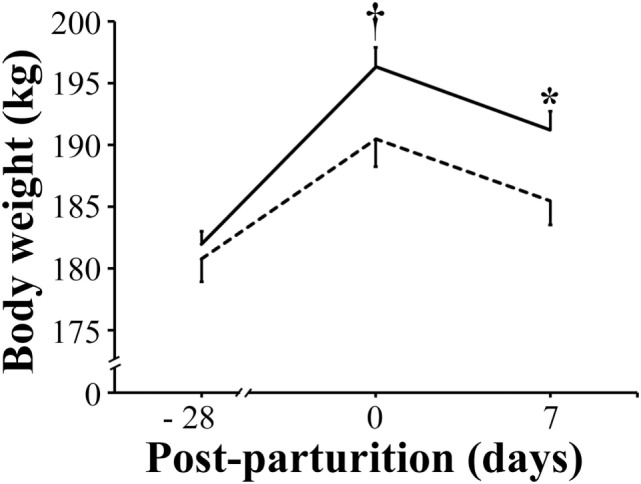
Mean body weight of sows. Solid bar: sows supplemented with probiotic compound and administered with a commercial live porcine epidemic diarrhea vaccine. Dashed line: control sows (vaccine only, no probiotics). * denotes significant differences between sow groups (*P* < 0.05). † denotes tendency of significance between sow groups. Values are mean ± SE.

In comparison with that of VC sows, the mean body weight of VP sows showed only a tendency (*P* < 0.1) to be greater as they approached parturition (191 vs 196 kg, respectively), but it was significantly higher (*P* < 0.05) by day 7 post-parturition (186 vs 191 kg, respectively) (Figure 2).

### Production Performance of Milk

**Figure 3:**
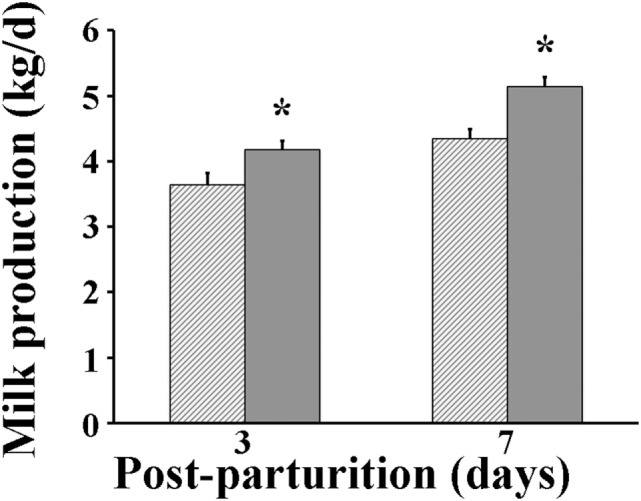
Milk production of sows. Hashed bars: control sows (vaccine only, no probiotics). Solid bars: sows supplemented with probiotic compound and administered with a commercial live porcine epidemic diarrhea vaccine. * denotes significant differences between sow groups (*P* < 0.05).

**Figure 4:**
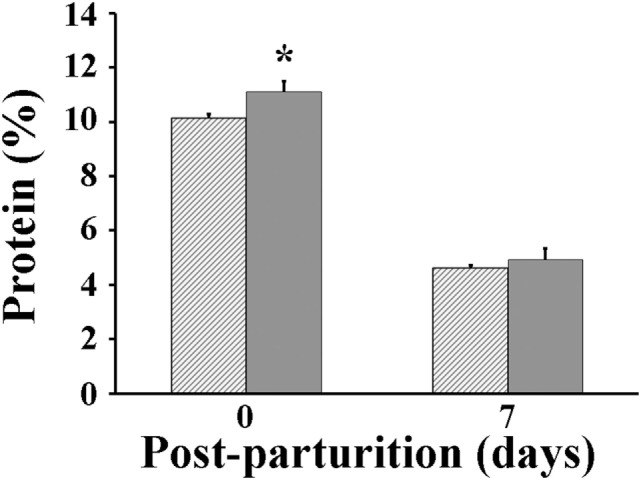
Total protein percentage in milk of sows. Hashed bars: control sows (vaccine only, no probiotics). Solid bars: sows supplemented with probiotic compound and administered with a commercial live porcine epidemic diarrhea vaccine. * denotes significant differences between sow groups (*P* < 0.05).

Following parturition, milk produced by VP sows was found to be significantly greater (*P* < 0.05) than that by VC sows at days 3 (4.18 vs 3.63 kg/day, respectively) and 7 after parturition (5.14 vs 4.34 kg/day, respectively) (Figure 3). The protein percentage in milk of VP sows was significantly greater (*P* < 0.05) at parturition day (11.1%) than that measured in milk of VC sows (10.1%) (Figure 4). However, when it was measured again at day 7 post-parturition, no difference in protein percentage was detected between milk samples of VP and VC sows (4.9 vs 4.6%).

### Lactogenic Immunity Parameters

**Figure 5:**
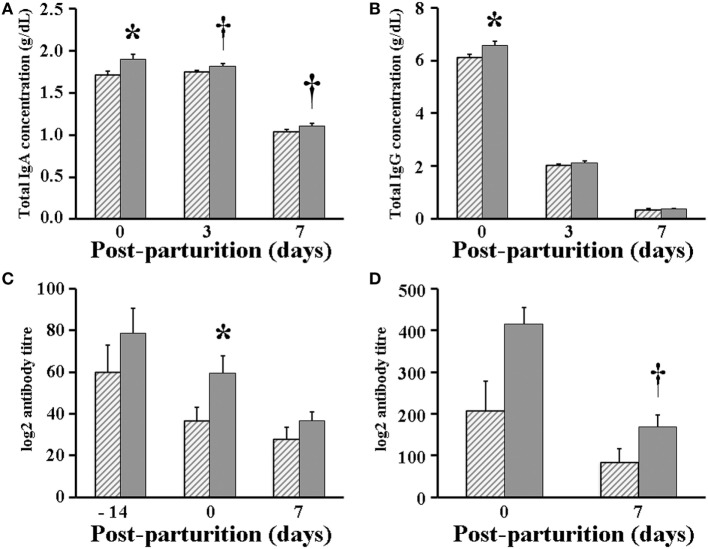
Immunology parameters measured in serum and whey of sows. **(A)** Total immunoglobulin (Ig)A concentration in whey. **(B)** Total IgG concentration in whey. **(C)** Total porcine epidemic diarrhea (PED) virus-specific antibody titer in serum. **(D)** Total PED virus-specific antibody titer in whey. Hashed bars: control sows (vaccine only, no probiotics). Solid bars: sows supplemented with probiotic compound and administered with a commercial live PED vaccine. * denotes significant differences between sow groups (*P* < 0.05). † denotes tendency of significance between sow groups. Values are mean ± SE.

When looking at the lactogenic immunity parameters, total IgA concentration at day 0 (parturition day) was significantly (*P* < 0.05) higher in whey of VP sows (1.90 g/dL) than in that of VC sows (1.72 g/dL), but afterward, total IgA concentration only showed a tendency (*P* < 0.1) to increase in whey of VP but not VC sows, when it was measured at days 3 (1.82 vs 1.75 g/dL, respectively) and 7 post-parturition (1.11 vs 1.04 g/dL, respectively) (Figure 5A). Similarly, the concentration of total IgG was significantly (*P* < 0.05) greater in whey of VP sows (6.58 g/dL) at day 0 when compared with that of VC sows (6.10 g/dL). However, unlike IgA, the concentration of IgG was the same in whey of both VP and VC sows, when it was measured at days 3 (2.12 vs 2.04 g/dL, respectively) and 7 post-parturition (0.36 vs 0.35 g/dL, respectively) (Figure 5B). In addition, while the antibody titer was found significantly (*P* < 0.05) higher at day 0 in serum of VP sows (59.7 in geographic mean) when compared with that of VC sows (36.8), it was similar in serum of sows in both experimental groups 14 days prior to parturition (78.8 vs 59.7, respectively) and 7 days post-parturition (36.8 vs 27.9, respectively) (Figure 5C). Likewise, the antibody titer was found to be similar (415.9 vs 207.9 at day 0; 168.9 vs 84.4 at day 7) in whey of both VP and VC sows (Figure 5D).

### Reproductive Performance

**Figure 6:**
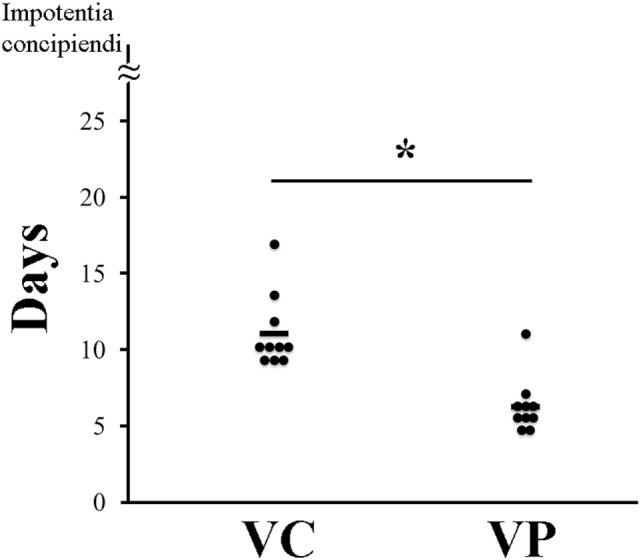
Recurrence of estrus in sows. VC: control sows (vaccine only, no probiotics). VP: sows supplemented with probiotic compound and administered with a commercial live porcine epidemic diarrhea vaccine. * denotes significant differences between sow groups (*P* < 0.05). Bars represent the mean number of days between weaning and return to estrus.

**Figure 7:**
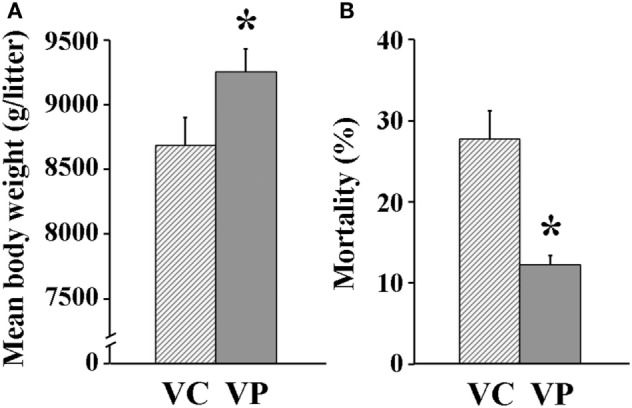
Reproductive parameters measured for sows. **(A)** Mean litter weight at birth (*n* = 10). **(B)** Mortality percentage of piglets during suckling (0–21 days post-parturition). Solid bars: sows supplemented with probiotic compound, and administered with a commercial live porcine epidemic diarrhea vaccine. * denotes significant differences between sow groups (*P* < 0.05). † denotes tendency of significance between sow groups. Values are mean ± SE.

The combined administration of probiotics and vaccine had a positive effect on reproductive performance. Indeed, the days between weaning and return to estrus for most VP sows were fewer (7 days) (*P* < 0.05) than those for VC sows (10 days) (Figure 6). Moreover, piglets farrowed by VP sows had a significantly (*P* < 0.05) higher weight (9,252 g/litter) (Figure 7A) and a lower (*P* < 0.05) mortality percentage (12%) than those farrowed by VC sows (8,686 g/litter and 28%, respectively) (Figure 7B).

## Discussion

Porcine epidemic diarrhea-vaccinated sows that were also supplemented with probiotics improved their feed intake, gained reasonable bodyweight during the period prior to parturition, and had only marginal weight loss by day 7 post-parturition than did sows that were vaccinated but did not receive probiotics supplementation (Figures 1 and 2). Moreover, those sows receiving both probiotic supplementation and vaccine administration produced more milk than did sows that were only vaccinated (Figure 3). Previously, Alexopoulos et al. (33) showed that supplementing feed with a probiotic compound containing *Bacillus licheniformis* and *Bacillus subtilis* spores not only increased the body weight of sows but also helped minimize their weight loss during the suckling period. Likewise, Kritas et al. (34) reported that supplementing with *B. subtilis* C-3102 resulted in higher feed consumption and better body condition of sows. Thus, it is very likely that in the present study there were similar effects, in which probiotics indirectly promoted weight gain in sows by competing with pathogens in the gut and stimulating the immune system of the host and hence making the animal more resistant to infections (35). It is also possible that probiotics fought back the viral infection directly and decreased the viral load in sows and eventually in piglets as Sirichokchatchawan et al. (28) recently suggested when discussing the potency of probiotic LAB to reduce PED infectivity *in vitro*. In addition, the peptide–zinc mixture added to the probiotic compound may have also contributed to enhance the immune response. Indeed, zinc has been previously shown to help reduce diarrhea incidence and increase body weight in pigs by stimulating the immune response against viral infection (36). Ultimately, healthier sows in the VP group consumed more feed, utilized better feed nutrients, and consequently gained more weight and produced more milk (Figures 3 and 4).

In commercial swine farms, pregnant sows are inoculated with vaccines to trigger an immunoprophylactic reaction known as lactogenic immunity (15). Lactogenic immunity protects piglets against infections *via* the suckling of colostrum and milk of vaccinated sows (17). Nonetheless, in the present work, probiotics supplementation significantly improved the concentration of total IgA and IgG in the milk of sows than did PED vaccination alone, possibly resulting from stimulation of gut immunity by bacteria in the administered probiotic compound (Figures 5A,B).

The sole administration of PED vaccine to sows has been shown to increase PED-specific IgG levels in serum (15). Interestingly, by administering the PED vaccine along with probiotics supplementation, we were able to trigger an even higher serum PED-specific antibody titer than did PED vaccination alone, very likely due to a greater stimulation of the immune system of sows. Then, as our results show, these antibodies were mobilized to fight back PED infection before and after parturition (Figure 5C), which consequently also helped to stimulate PED-specific antibodies production in whey (Figure 5D). Finally, once piglets started to suckle, passive immunity against PED was putatively transferred from sows *via* colostrum and milk (17, 18), possibly inducing early maturation of gut immunity of piglets that lessened the infection, which in turn heightened the effect of vaccination (37). This ultimately resulted in the farrowing of piglets with a greater weight and a lower mortality percentage by sows receiving both probiotics and the PED vaccine (Figure 7).

Viral infections can cause serious biological disturbances in sows including a higher recurrence of estrus (12), as well as abnormal tissue development such as ovarian cysts formation (38). These disorders can lead to low fertility (12). In addition, it is believed that a decrease in appetite and lower efficiency of nutrient utilization during the course of an infection can cause a lower body weight of sows which in turn cause a longer period before sows can return to estrus (39). Nonetheless, it has been previously shown that *B. subtilis* C-3102 supplementation helped reduce the number of days between weaning and return to estrus (34). Furthermore, a probiotic compound containing the same bacteria used in this study improved the percentage of sows returning to estrus (29). In agreement with this, in the present work PED vaccination and probiotic administration to PED-infected sows considerably lowered the number of days of recurrence of estrus than did PED vaccination alone (Figure 6). As previously mentioned, it is very likely that an enhanced microbiota permitted sows to eat more and utilized nutrients more efficiently, thus preventing malnutrition from causing a longer period between weaning and return to estrus (39).

Olanratmanee et al. (12) found that PED infection caused a reduction in the body weight of piglets at birth. In the present study, piglets farrowed by sows vaccinated against PED and supplemented with probiotics had a greater mean litter weight at birth and a lower mortality percentage than did those farrowed by sows receiving PED vaccination but no probiotic supplementation (Figures 7A,B). However, unlike our study, Kritas et al. (34) found that supplementation of a single probiotic strain (*B. subtilis* C-3102) to sows infected with pathogenic *E. coli* did not alter the body weight at birth of piglets. This apparent discrepancy may be due to in the present study and unlike that by Kritas et al. a probiotic mixture was given to sows. Indeed, it has been demonstrated both *in vivo* and *in vitro* that multistrain probiotic compounds are more effective at inhibiting pathogens than single-strain probiotics and that some strain mixtures are more effective than others (40, 41).

In conclusion, we demonstrated that supplementation of a mixture of probiotics and a peptide–zinc compound enhanced the immune system of PED virus-infected sows. In addition, we also showed that probiotics were able to act as adjuvant to commercial live PED vaccines administered to pregnant sows.

## Ethics Statement

This experiment was approved by the ethical committee of Inatomi Animal Hospital in Japan. Takio Inatomi belongs to Inatomi Animal Hospital informed consent to animal owners.

## Author Contributions

TI designed and performed this experiment and contributed to the discussion and interpretation of data. MA supplied the experimental materials and contributed to the discussion and interpretation of data. GR-P wrote the manuscript. RI contributed to the discussion and interpretation of data and wrote the manuscript. TT designed this experiment, contributed to the discussion and interpretation of data, and wrote the manuscript. All authors have read and approved the final manuscript.

## Conflict of Interest Statement

TI is the president of a commercial animal hospital namely “Inatomi Animal Hospital.” MA is employed by Toa Pharmaceutical Co., Ltd. GR-P and TT is employed by Kyoto Institute of Nutrition & Pathology, Inc. All remaining authors have no conflicts to disclose.

## Footnotes

^1^http://www.jp-nisseiken.co.jp/en/products/pdf/pig/PED_en.pdf.

^2^http://www.jppa.biz.
